# Surgical Management of Complicated Chronic Uveitis: A Case Report

**DOI:** 10.22336/rjo.2025.71

**Published:** 2025

**Authors:** Andreea Alexandra Mihaela Muşat, Nicoleta Zamfiroiu-Avidis, Cornel Ştefan, Andreea Schmitzer, Gabriela Muşat, Gabriela Udrea, Ioana Stella Popescu, Ovidiu Muşat

**Affiliations:** *“Carol Davila” University of Medicine and Pharmacy, Bucharest, Romania; 2 Clinical Emergency Eye Hospital, Bucharest, Romania; 3 “Dr. Carol Davila” Central Military Emergency University Hospital, Bucharest, Romania; 4 Apel Laser SRL, Ilfov, Romania

**Keywords:** chronic uveitis, uveitic glaucoma, complicated cataract, vitrectomy

## Abstract

**Background:**

Chronic uveitis is a common vision-threatening condition that can lead to many complications and may not respond to conventional medical therapy. This case report aims to emphasize the need for a personalized approach to these challenging cases to reestablish visual function.

**Case presentation:**

A 29-year-old female patient, C.F., developed bilateral anterior uveitis of unknown origin that evolved into a chronic, refractory form. Topical, as well as systemic therapy, failed to control the inflammation, leading to complications in the form of cataract and secondary glaucoma. Surgical management involved Ahmed valve implantation in both eyes and pars plana vitrectomy.

**Discussion:**

This particularly challenging case highlights the complex challenges in managing uveitis of unknown etiology, especially when complicated by secondary glaucoma. In young patients, progressive structural changes due to inflammation can rapidly lead to permanent vision loss. A combination of surgical procedures resulted in favorable anatomical and functional outcomes; however, long-term follow-ups remain essential to monitor for recurrent inflammation, intraocular pressure fluctuations, and other potential complications.

**Conclusion:**

This case highlights the challenges and the need for a multidisciplinary approach in chronic, treatment-resistant uveitis with secondary complications.

## Introduction

Uveitis was historically referred to as a range of inflammatory processes affecting the uveal tract (iris, ciliary body, and choroid); however, any part of the eye can be affected. It can be further classified as anterior, intermediate, posterior, or panuveitis depending on the anatomical site of the most involved location [1]. The etiology can range from infections, inflammatory diseases, and trauma, to idiopathic causes [2]. The idiopathic causes of uveitis are responsible for a significant percentage of all cases, about 48 to 70% [3]. The young population is primarily affected, resulting in substantial economic and social burdens [4].

Anterior uveitis is defined as the inflammation of the anterior uveal tract (iris, ciliary body). The acute form is the most common type, and it typically lasts less than 3 months [5].

Although it can be self-limiting in some cases, it can also lead to a chronic form [6]. Symptoms may vary, covering a spectrum of complaints such as decreased visual acuity, headache, photophobia, and conjunctival hyperemia [7]. Chronic anterior uveitis is defined as the inflammation of the anterior uvea that lasts longer than 3 months [5].

Establishing the correct diagnosis is crucial to ensure the patient receives the appropriate and efficient therapy. Understanding the etiology of the inflammatory process requires a comprehensive approach, including history taking, ocular and systemic evaluation, and a response to treatment that provides key information [8]. In the absence of adequate therapy, severe complications such as glaucoma, cataract, corneal opacity, and macular edema may result in a decline in visual acuity [9].

## Case presentation

### Patient information

Patient C.F., female, age 29 years old, had a history of diagnosed anterior chronic uveitis in both eyes since 2018 (right eye) and 2020 (left eye), for which she was under treatment with topical dexamethasone and tropicamide and systemic prednisone and sulfasalazine and underwent multiple subconjunctival injections with dexamethasone. She also had a history of episodic joint pain in the right hip and non-specific rash, but no past relevant medical history.

### Clinical findings

The patient had a history of a rapid onset of the disease with symptoms that included ocular pain, redness, blurred vision, and photophobia. When she was admitted to our service, the best corrected visual acuity (BCVA) on presentation was 0.1 with correction on the right eye and 0.6 with correction on the left eye. Slit lamp examination in both eyes revealed a clear cornea, no cells or flare in the anterior chamber, anterior and posterior synechiae on 360 degrees with seclusion pupillae and complicated cataract. Fundus examination showed that the retina was attached with no inflammatory findings, and the cup disc ratio was 0.8. Intraocular pressure (IOP) measured with a non-contact tonometer was 44 mmHg in the right eye (OD) and 41 mmHg in the left eye (OS).

### Diagnostic Assessment

An extensive workup was performed in collaboration with the internal medicine and rheumatology departments, which included blood tests assessing autoimmune and infectious diseases, hepatic and renal function, complete blood count, inflammatory markers, and imaging examinations. It was revealed that HLA B08 and HLA B35 were positive, both of which are associated with autoimmune disease and susceptibility to certain infections, but neither was specific [9-20].

Magnetic resonance imaging (MRI) of the pelvis showed no signs of sacroiliitis, but identified a subtle, superficial peritrochanteric bursitis bilaterally, more marked on the right side, with a normal echography. No infection markers were positive. The differential diagnosis included uveitis of different causes. Still, since the initial panel of tests was non-conclusive and no definitive cause was found, the diagnosis of idiopathic chronic uveitis was established.

### Therapeutic intervention

**Fig. 1:**
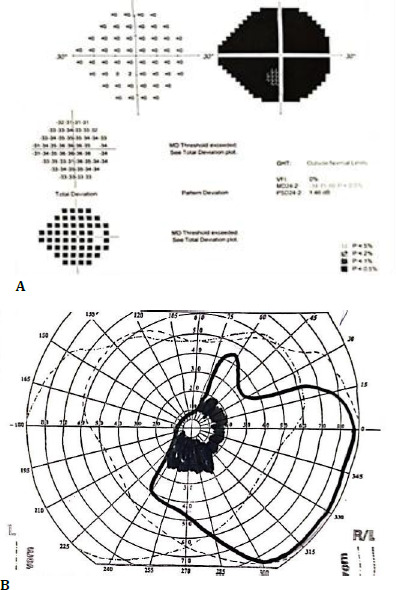
**A, B** Humphrey visual field test in OD (right) showing total scotoma and Goldmann visual field test in OS (left) showing a dense central scotoma and a constricted field, suggesting advanced glaucoma

Initial medical management included anti-glaucomatous therapy with topical dorzolamide, timolol, latanoprost, and brimonidine, and anti-inflammatory treatment with topical and systemic corticosteroids. Because the medical therapy failed to control the IOP, YAG-laser peripheral iridotomies were performed adequately in both eyes, and an Ahmed Valve was implanted in the right eye. Following a period of stable disease, a flare-up occurred, leading to the commencement of systemic methotrexate therapy. As the cataract continued to advance, the BCVA decreased to hand motion in OD and 0.4 in OS with an IOP within normal limits and no signs of inflammation in both eyes. Visual field tests were performed, as shown in **Fig. 1A, B**. The fundus was not visible due to the advanced cataract, and the B-scan echography revealed vitreous echoes, likely of inflammatory origin, with an attached retina in both eyes. She was then admitted to our service, where phacoemulsification with posterior chamber intraocular lens (IOL) implantation, anterior and posterior synechiolysis, peripheral iridectomies, posterior capsulotomy, and posterior vitrectomy were performed. Intraocular injection of anti-VEGF was performed for the prevention of macular edema, and a parabulbar injection of triamcinolone was administered. The same surgical procedure was performed a month later in the left eye. As the IOP continued to rise in OS, an Ahmed Valve was implanted. No signs of retinal inflammation were observed in either eye.

### Follow-up and outcomes

Postoperatively, the inflammation was well controlled, and the intraocular pressure remained within normal limits under treatment with dorzolamide and timolol. At three months postoperative, BCVA was 0.1 in OD and 0.7 in OS. No intraoperative or postoperative complications were reported.

**Fig. 2:**
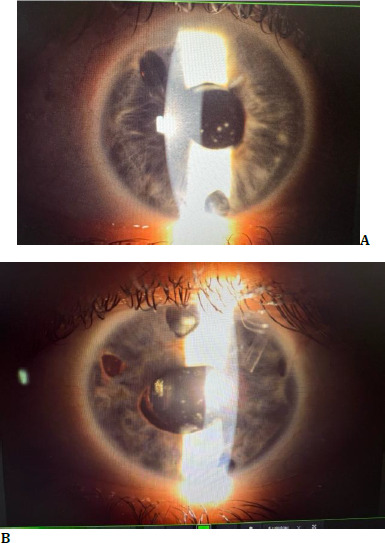
**A, B** Anterior segment of OD (right) and OS (left) showing patent peripheral iridotomies, Ahmed Valves, IOLs in posterior chambers, and no signs of inflammation

**Fig. 3:**
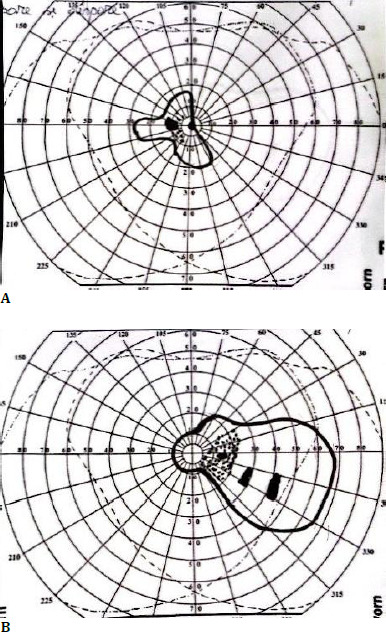
**A, B** Goldmann visual field tests in OD (right) and OS (left) indicate severe peripheral field loss

At six months postoperatively, the patient independently decided to discontinue the methotrexate therapy, which led to a significant decline in visual acuity - hand motion in OD and 0.05 in OS due to inflammatory deposits on the surface of the IOLs. The deposits were cleared successfully using YAG laser treatment. Following the reintroduction of immunosuppressive therapy, BCVA improved to 0.5 OD and 1 OS. **Fig. 2A, B** presents the anterior segment of both eyes. Even though the BVCA improved, and the intraocular inflammation resolved, the visual fields performed in both showed marked loss of peripheral vision due to the long-standing glaucoma, as observed in **Fig. 3A, B**.

## Discussion

Managing cases of uveitis of unknown origin can be challenging, especially when they are non-responsive to traditional medical therapy. Surgical intervention in uveitis-related glaucoma should be considered when the maximal anti-glaucomatous treatment fails to control the IOP adequately. The benefit of using Ahmed Valves (AVs) in young patients with secondary uveitic glaucoma lies in the device's capacity to provide a sustained IOP reduction in eyes with complex pathology, where conventional filtration surgery can sometimes fail due to aggressive healing responses and sustained inflammation [21]. AVs are advantageous in cases with an increased risk of postoperative scarring, such as those involving uveitis. However, their use is not without risk. Long-term complications, such as device exposure, erosion, hypotony, hyphema, tube obstruction, and corneal decompensation, require close postoperative monitoring [22].

Pars plana vitrectomy plays numerous roles in the management of chronic uveitis [23]. Beyond its benefits in removing vitreous opacities, it also facilitates the visualization of the posterior pole intraoperatively. It enables the assessment and treatment of the possible uveitis-related complications, such as cystoid macular edema, epiretinal membranes, retinal detachment, optic neuropathy, and retinal vascular occlusion [24].

Chronic uveitis, especially when the underlying cause is unclear or unknown, requires a multidisciplinary approach to ensure adequate diagnosis and treatment. The collaboration between the ophthalmologist and the rheumatologist, internal medicine specialist, or infectious disease specialist is crucial in the management of systemic diseases that may not be apparent on initial ophthalmologic assessment. Although no definitive underlying disease was identified in this case, coordinated care played a crucial role in prescribing immunosuppressive medication that stabilized the inflammation and prevented vision-threatening sequelae.

## Conclusion

Chronic uveitis, especially cases complicated with cataract and secondary glaucoma, can present significant therapeutic challenges and may require complex surgical intervention. Early referral and timely surgical planning are crucial when the response to medical therapy proves to be unsatisfactory. Long-term follow-ups and close monitoring are essential to maintaining IOP control, minimizing inflammation, and preserving visual function. In addition to the health care provider’s role, the patient also has a great responsibility in respecting the doctor’s recommendations to achieve the best possible results.
